# The Synergistic Immunoregulatory Effects of Culture-Expanded Mesenchymal Stromal Cells and CD4^+^25^+^Foxp3^+^ Regulatory T Cells on Skin Allograft Rejection

**DOI:** 10.1371/journal.pone.0070968

**Published:** 2013-08-05

**Authors:** Jung Ho Lee, Eun-Joo Jeon, Nayoun Kim, Young-Sun Nam, Keon-Il Im, Jung-Yeon Lim, Eun-Jung Kim, Mi-La Cho, Ki Taik Han, Seok-Goo Cho

**Affiliations:** 1 Laboratory of Immune Regulation, Convergent Research Consortium for Immunologic Disease, Seoul St. Mary’s Hospital, The Catholic University of Korea College of Medicine, Seoul, Korea; 2 Department of Plastic and Reconstructive Surgery, The Catholic University of Korea College of Medicine, Seoul, Korea; 3 Rheumatism Research Center, Catholic Institutes of Medical Science, Seoul, Korea; 4 Catholic Blood and Marrow Transplantation Center, Seoul St. Mary’s Hospital, The Catholic University of Korea College of Medicine, Seoul, Korea; Children’s Hospital Boston, United States of America

## Abstract

Mesenchymal stromal cells (MSCs) are seen as an ideal source of cells to induce graft acceptance; however, some reports have shown that MSCs can be immunogenic rather than immunosuppressive. We speculate that the immunomodulatory effects of regulatory T cells (Tregs) can aid the maintenance of immunoregulatory functions of MSCs, and that a combinatorial approach to cell therapy can have synergistic immunomodulatory effects on allograft rejection. After preconditioning with Fludarabine, followed by total body irradiation and anti-asialo-GM-1(ASGM-1), tail skin grafts from C57BL/6 (H-2k^b^) mice were grafted onto the lateral thoracic wall of BALB/c (H-2k^d^) mice. Group A mice (control group, n = 9) did not receive any further treatment after preconditioning, whereas groups B and C (n = 9) received cell therapy with MSCs or Tregs, respectively, on days −1, +6 and +13 relative to the skin transplantation. Group D (n = 10) received cell therapy with MSCs and Tregs on days −1, +6 and +13. Cell suspensions were obtained from the spleens of five randomly chosen mice from each group on day +7, and the immunomodulatory effects of the cell therapy were evaluated by flow cytometry and real-time PCR. Our results show that allograft survival was significantly longer in group D compared to the control group (group A). Flow cytometric analysis and real-time PCR for splenocytes revealed that the Th2 subpopulation in group D increased significantly compared to the group B. Also, the expression of Foxp3 and STAT 5 increased significantly in group D compared to the conventional cell therapy groups (B and C). Taken together, these data suggest that a combined cell therapy approach with MSCs and Tregs has a synergistic effect on immunoregulatory function *in vivo*, and might provide a novel strategy for improving survival in allograft transplantation.

## Introduction

Composite tissue allotransplantation (CTA), in which composite tissue consisting of skin, muscle and bone is transferred from one person to another, has successfully been used to treat tissue loss resulting from trauma, tumors, or congenital deformity. The first successful human hand transplant was performed in 1998, and the first successful partial face allotransplant was performed in 2005 [1], [2].

Nonspecific immunosuppressive drugs such as tacrolimus, mycophenolate mofetil and steroids have been used in patients receiving CTA, as in solid organ transplantation, with excellent results [1], [3], [4]. However, since the aim of surgery is to improve quality of life, and life-long administration of immunosuppressive agents can have fatal sequelae, researchers have been seeking alternative ways to induce tolerance [5], [6], [7], [8].

One strategy currently being considered is the use of tolerance-inducing cells, such as mesenchymal stromal cells (MSCs) and regulatory T cells (Tregs), because they can reduce the side effects caused by traditional immunosuppressants [9], [10], [11], [12]. MSCs not only have multilineage differentiation potential but also possess remarkable immunosuppressive properties, which can inhibit the proliferation and function of major immune cells, including T cells, B cells and natural killer cells [13], [14], [15]. It has also been shown that donor MSCs are able to inhibit T-cell proliferation in a mixed lymphocyte culture, preventing graft-*versus*-host disease (GVHD) as a result of a bone marrow transplant (BMT), and prolonging skin allograft survival in baboons [16], [17], [18].

However, it also has been shown that MSCs can be immunogenic rather than immunosuppressive in a skin allograft model of immunocompetent host and bone marrow transplants in a non-myeloablative setting [19], [20]. Therefore, the paradigm that MSCs are immunoprivileged has shifted to the point where they can be considered immunogenic under specific inflammatory conditions [21].

Taking this into account, we speculate that the immunomodulatory effects of Tregs in allotransplantation can help to maintain MSC immunoregulatory functions, and that combination cell therapy using both MSCs and Tregs can have synergistic immunomodulatory effects on allograft rejection. We investigated both whether combination cell therapy could prolong skin allograft survival compared to conventional cell therapy, and the underlying mechanisms.

## Materials and Methods

### Mice

Eight-to-ten-week-old C57BL/6 (H-2k^b^) and BALB/c (H-2k^d^) mice were purchased from OrientBio (Sungnam, Korea). The mice were maintained under specific pathogen-free conditions in an animal facility with controlled humidity (55±5%), light (12/12h light/dark), and temperature (22±1°C). The air in the facility was passed through a HEPA filtration system designed to exclude bacteria and viruses. Animals were fed mouse chow and tap water *ad libitum*. The protocols used in the present study were approved by the Animal Care and Use Committee of The Catholic University of Korea (Permit Number: 2010-0204-02).

### Isolation and Culture of MSCs

MSCs were generated as described previously [22]. Briefly, donor (C57BL/6, H-2k^b^) bone marrow cells were collected by flushing mouse femurs and tibias with Dulbecco’s modified Eagle’s medium (Gibco, Carlsbad, CA, USA) supplemented with 15% heat-inactivated fetal bovine serum (FBS) (Gibco, Carlsbad, CA, USA). Suspended cells were plated onto 95-mm culture dishes in 1 ml of complete medium, at a density of 2×10^7^/ml. Cultures were incubated at 37°C with 5% CO_2_ in a humidified chamber. After 3 h, nonadherent cells were removed by changing the medium. When cells were 80% confluent, they were trypsinized by incubation in 0.5 ml of 0.25% trypsin/1 mM ethylenediaminetetraacetic acid for 2 min at room temperature. Trypsin was neutralized by the addition of 1.5 ml of complete medium. Cells were harvested and expanded in 75-T flasks, and cultures were maintained at 37°C with 5% CO_2_ in a humidified chamber and subcultured before confluency. After 10 passages, the MSCs were surface-stained for c-kit (2B8, 0.5 µg/ml, BioLegend), Rat IgG2b (RTK4530, 0.5 µg/ml, BioLegend), CD11b (M1/70, 0.2 µg/ml, BD Pharmingen), Rat IgG2b (A95-1, 0.2 µg/ml, BD Pharmingen), CD34 (MEC14.7, 0.2 µg/ml, BioLegend), Rat IgG2a (RTK2758, 0.2 µg/ml, BioLegend), CD106 (429, 0.5 µg/ml, BD Pharmingen), Rat IgG2a (R35–95, 0.5 µg/ml, BD Pharmingen), CD45 (30-F11, 0.2 µg/ml, BD Pharmingen), CD31 (MEC13.3, 0.2 µg/ml, BD Pharmingen), Sca-1 (D7, 0.2 µg/ml, BioLegend), CD44 (IM7, 0.5 µg/ml, eBioscience), Rat IgG2b (eB149/10H5, 0.5 µg/ml, eBioscience), CD29 (HMβ1-1, 0.5 µg/ml, BioLegend), and Armenian Hamster IgG (HTK888, 0.5 µg/ml, BioLegend) and were then characterized by flow cytometry. Previous to surface staining, MSCs were Fc-blocked with CD16/CD32 (2.4G2, 1 µg/ml, BD Pharmingen) for 15 minutes at room temperature. After blocking, 1 µl of each antibody was added to cells and incubated for 30 minutes at room temperature. Any unbound antibodies were removed by washing the cells in flow cytometry staining buffer.

### Generation and Characterization of Tregs

Tregs were generated as described previously [23]. Briefly, spleen cells from the C57BL/6 mice were incubated with magnetic microbeads conjugated to mouse CD4 monoclonal antibody (L3T4 MicroBeads; Miltenyi Biotec, Germany) for 15 min at 4°C, rinsed and placed on a miniMACS column. After isolation, the cells were counted and assessed for viability. Cells were cultured in RPMI 1640 medium supplemented with 5% heat-inactivated FBS (Gibco, Carlsbad, CA, USA). CD4^+^ T cells were activated with plate-bound anti-CD3 (1 µg/ml; BD PharMingen) and soluble anti-CD28 (1 µg/ml; Biolegend) in 12-well plates (1×10^6^ cells/well). The culture medium was supplemented with all-trans-retinal (1 µM; Sigma-Aldrich) and TGF-β (5 ng/ml; PeproTech) for conversion. Cells were incubated for 72 h at 37°C and 5% CO_2_ and were then collected and characterized by flow cytometry after staining for CD4 (RM4–5, 0.2 µg/ml, eBioscience,), CD25 (PC61, 0.1 µg/ml, BioLegend), Foxp3 (FJK-16s, 0.4 µg/ml, eBioscience), CD62L (MEL-14, 0.25 µg/ml, BioLegend), CTLA-4 (UC10-4B9, 0.2 µg/ml, BioLegend), GITR (DTA-1, 0.5 µg/ml, eBioscience), PD-1 (J43, 0.2 µg/ml, BD Pharmingen), CD103 (2E7, 0.2 µg/ml, BioLegend), ICAM-1(YN1/1.7.4, 0.5 µg/ml, eBioscience), ICOS (C398.4A, 0.5 µg/ml, eBioscience) and CD 44 (IM7, 0.5 µg/ml, eBioscience).

### Experimental Design

We used a skin allograft model to evaluate the effects of the cell therapy. Full thickness tail skin grafts (1×1 cm^2^) from donor mice (C57BL/6) were grafted on the lateral flank of recipient mice (BALB/c). Graft sites were protected under sterile gauze and monitored daily from day +5 by visual inspection. Graft rejection was defined as the complete destruction or desiccation of the grafted skin on inspection. Graft survival curves were drawn using the Kaplan-Meier method, and graft survival between two groups was compared using the log-rank test.

Group A (n = 9), the control group, received 100 mg/kg of Fludarabine (Baxter oncology GmbH, Germany) intraperitoneally from day −8 to day −4, preconditioning irradiation (400 cGy) on day −3, and 100 µl anti-ASGM-1 (WaKo, Japan) intraperitoneally on day −2.

Group B (n = 9) was treated using the same protocol as group A, but also received a 1×10^6^ dose of MSCs intraperitoneally (days −1, +6, +13). Group C (n = 9) was treated using the same protocol as group A, but also received a 2×10^6^ dose of Tregs intravenously (days −1, +6, +13). Group D (n = 10) was treated using the same protocol as group A, but also received a 1×10^6^ dose of MSCs intraperitoneally and a 2×10^6^ dose of Tregs intravenously (days −1,+6,+13).

### Flow Cytometric Evaluation of T cell Repopulation

To ascertain the *in vivo* immunoregulatory mechanisms of combination cell therapy, we investigated changes in the T-cell subpopulation after treatment. On day +7, spleens from recipient mice (n = 5 in each group) were harvested from each group and flow cytometry was performed using various combinations of fluorochrome-conjugated antibodies to CD4 (RM4–5, 0.2 mg/ml, eBioscience), CD25 (PC61, 0.2 mg/ml, BioLegend), Foxp3 (FJK-16s, 0.2 mg/ml, eBioscience), IFN-γ (XMG1.2, 0.2 mg/ml, eBioscience), and IL-4 (11B11, 0.2 mg/ml, BD Pharmingen). Cytokine secretion was stimulated by PMA (25 ng/ml; Sigma-Aldrich) and ionomycin (250 ng/ml; Sigma-Aldrich) in the presence of Golgi-stop (1 µl/ml; BD Bioscience) in 5% CO_2_ at 37°C for 4 hours. A total of 1×10^6^ spleen cells were washed and re-suspended in FACS buffer (phosphate-buffered saline, 0.5% bovine serum albumin, 0.1% sodium azide). Total spleen cells were washed and stained with primary (surface) fluorochrome-conjugated antibodies. Cells were then incubated for another 30 min at 4°C with antibodies and washed twice with FACS buffer. Spleen cells were fixed and permeabilized using the BD Cytofix/Cytoperm Kit (BD Bioscience), and then stained with intracellular antibodies. The Foxp3 Staining Buffer Set (eBioscience) was used for Foxp3 staining. The stained cells were resuspended in FACS buffer, data were acquired using a FACS Calibur (BD Diagnostic System, Sparks, MD) and analyzed with the Flowjo software (TreeStar, San Carlos, CA).

### Real-time Quantitative PCR

We also performed real-time quantitative PCR to evaluate Foxp3 and STAT5 expression. Total RNA was extracted from 1×10^6^ splenocytes harvested on day +7 from recipient mice (n = 5 in each group), using TRIzol (Invitrogen). Chloroform (0.2 ml; Sigma-Aldrich) was added for every 1 ml of TRIzol used. Extracted RNA was shaken vigorously for 15 sec and incubated at room temperature for 2 min. After transferring the aqueous phase to a clean tube, isopropanol (Sigma-Aldrich) was added, followed by incubation at room temperature for 5 min. The RNA pellet was washed with 1 ml of 75% ethanol. After air-drying the RNA pellet for 10 min, it was dissolved in 12 µl of water and incubated at 55°C for 10 min. RNA preparations were treated with DNase I (according to the standard protocol) to remove genomic DNA. cDNA was synthesized by incubating 20 µl of mRNA in a sprint C1000 terminal cycler (Bio-rad). Negative controls contained all the elements of the reaction mixture except for template DNA. For quantification, relative mRNA expression of specific genes was obtained by the 2^−ΔCt^ method, using β-actin for normalization. The following gene-specific primers (5′→3′) were used: β-actin (forward; GAA ATC GTG CGT GAC ATC AAA G, and reverse; TGT AGT TTC ATG GAT GCC ACA G); Foxp3 (forward; GGC CCT TCT CCA GGA CAG A, and reverse; GCT GAT CAT GGC TGG GTT GT); STAT5 (forward; ATT ACA CTC CTG TAC TTG CGA, and reverse; GGT CAA ACT CGC CAT CTT GG). Diluted cDNA (10 µl) was mixed with 2 µl of primer and 10 µl IQ SYBR Green SuperMix, and was then assayed in triplicate on a CFX-96 real-time system (Bio-Rad) under the following conditions: denaturation at 95°C for 3 min, annealing for 30 sec at 58°C for β-actin and Foxp3, and at 58.4°C for STAT5, followed by 30 sec of extension at 72°C.

### Statistical Analysis

Data are presented as the mean ± SEM (standard error of mean). Statistical analysis was performed using Graphpad Prism (v.5.01). The comparisons between groups were analyzed statistically using the Kruskal-Wallis test. Pairwise group comparisons used the Mann-Whitney U test and p values were adjusted for multiple comparisons using Bonfferroni’s method to determine the statistical significance of these comparisons. Graft survival between groups was compared by Kaplan-Meier analysis and the log-rank test. A value of *p*<0.05 was considered to indicate statistical significance.

## Results

### Phenotypes of Culture-expanded MSCs and Tregs

**Figure 1:**
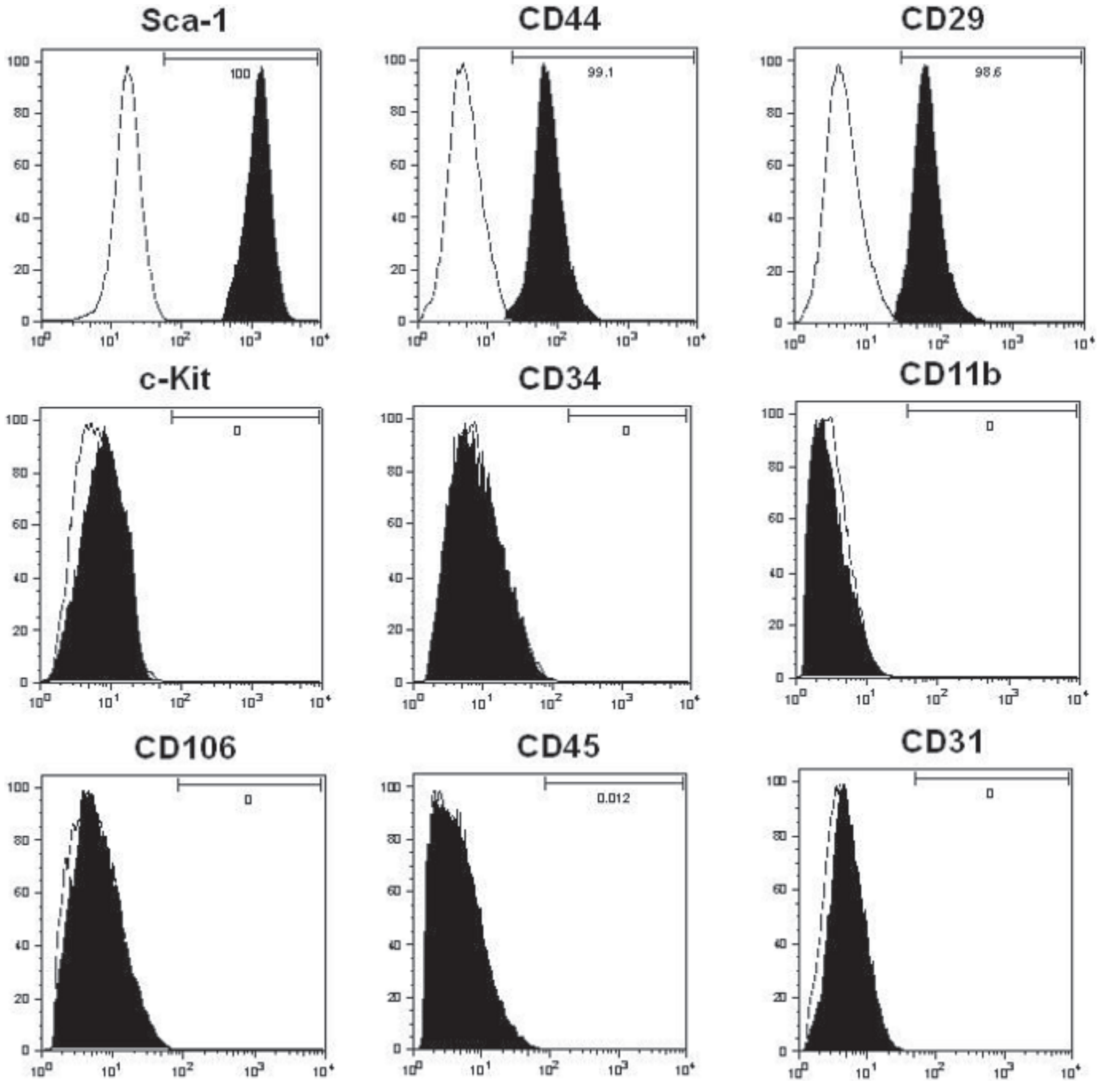
Immunophenotypes of *ex-vivo* expanded MSCs. Expanded MSCs are distinguished from hematopoietic cells by being negative for the expression of the cell-surface markers c-kit, CD11b, CD34, CD106, CD45, CD31 and positive for Sca-1, CD44 and CD29. White peaks indicate the isotype, black peaks indicate the phenotype antibody.

**Figure 2:**
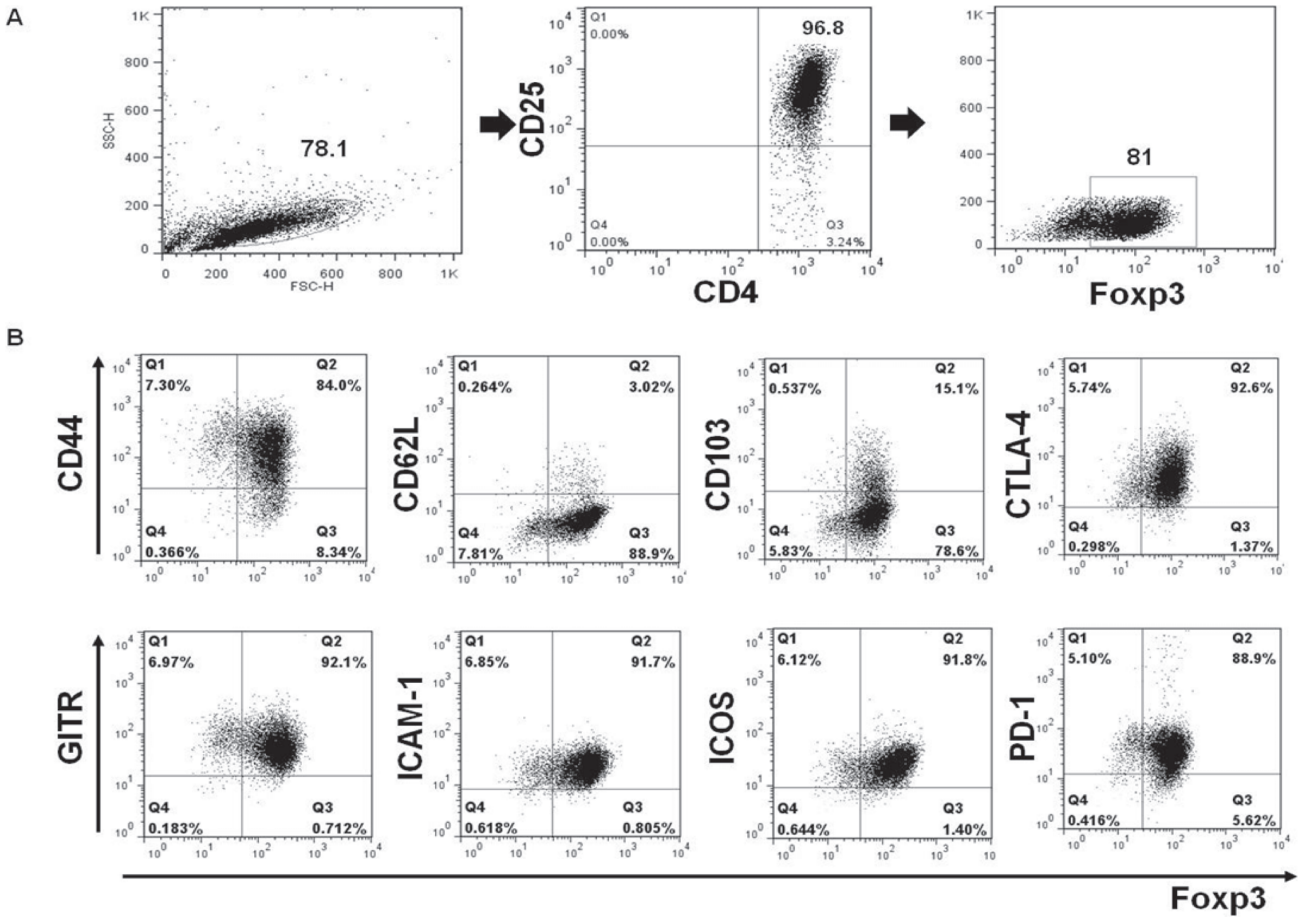
Immunophenotypes of *ex-vivo* expanded retinal-induced Tregs. (A) Retinal-induced CD4^+^CD25^+^ Tregs showed >96% purity on flow cytometry. (B) Tregs generated were characterized by positive expression of intracellular Foxp3, CTLA-4, and surface expression of the indicated markers (PD-1, GITR, ICAM-1, CD44, ICOS) in the gated T-cell populations. Also, they showed weak positive surface staining for CD62L and CD103. The percentages indicate numbers of double-positive cells.

Culture-expanded MSCs showed a typical fibroblast-like morphology and were uniformly positive for Sca-1, CD44, and CD29, but negative for c-Kit, CD11b, CD34, CD106, CD45, and CD 31(Fig. 1). Retinal-induced CD4+CD25+ Tregs showed >96% purity on flow cytometry and positive surface staining for several phenotypic Treg markers, including CD44, 1–3glucocorticoid-induced tumor necrosis factor receptor (GITR), intercellular adhesion molecule-1 (ICAM-1), inducible costimulator (ICOS) and programmed death-1 (PD-1). Also, they showed weak positive surface staining for CD62L and CD103 (Fig. 2).

### Effects of Cell Therapy on Skin Allograft Survival

**Figure 3:**
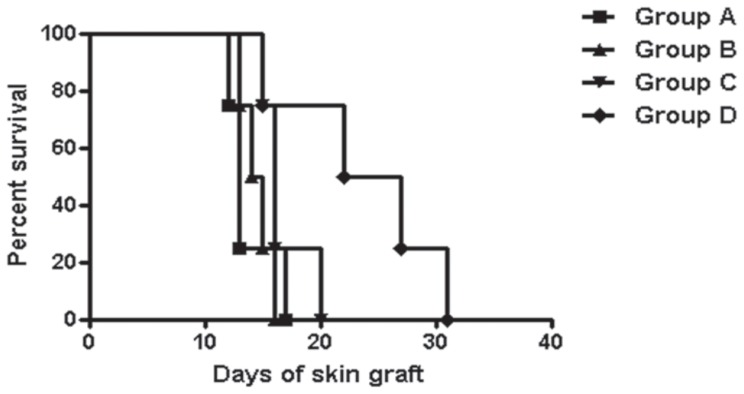
Skin allograft survival. Compared to the control group, only the combination cell therapy significantly prolonged graft survival (*p* = 0.028).

The median survival time for each group was as follows; group A, 13 days; group B, 14.5 days; group C, 16 days; and group D, 24.5 days (Fig. 3). Only the combination cell therapy significantly prolonged skin graft survival compared to the control group (*p* = 0.028).

### Effects of Cell Therapy on T-cell Repopulation

**Figure 4:**
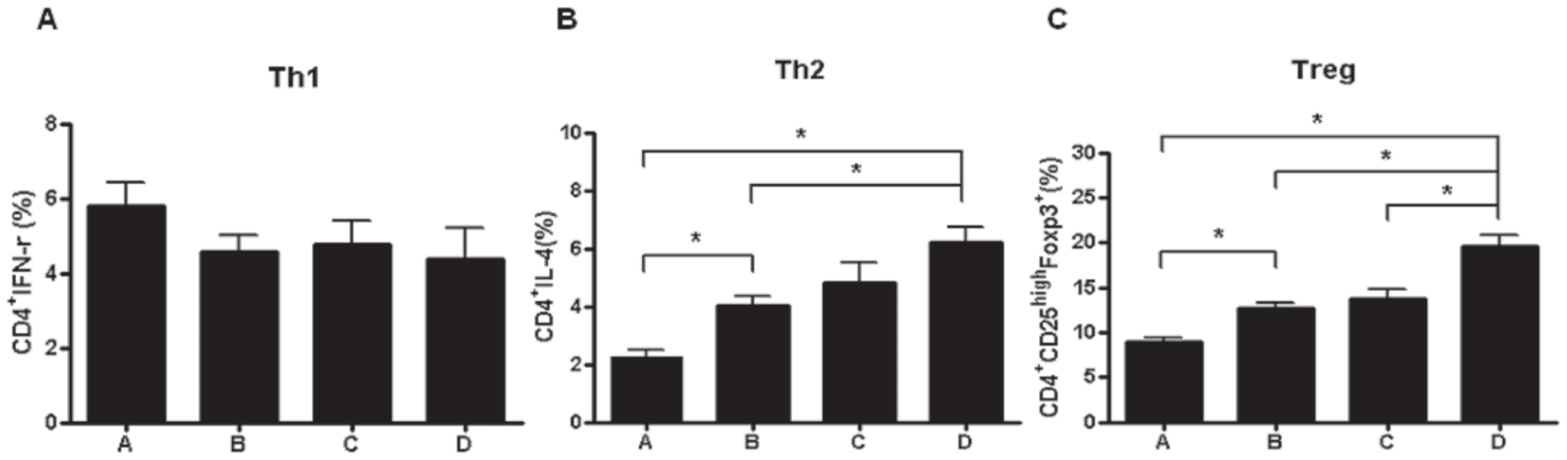
Effects of cell therapy on T-cell repopulation. (A) There was no significant difference in Th1 populations among the experimental groups. (B) Th2 populations were increased significantly in the combination cell therapy group compared to the control and MSC-treated groups. (C) Treg populations in the combination cell therapy group were increased significantly compared to other groups. The percentages in the axis of these figures are of CD4+ T cells. *p<0.05.

Flow cytometric analysis performed on day +7 showed no significant change in Th1 population in the experimental groups (Fig. 4). However, the Th2 population was significantly higher in the combination cell therapy group compared to the control and MSC-treated (*p*<0.05) groups. The total Treg population in the combination cell therapy group increased significantly compared to the control and MSC-treated and Tregs treated groups (*p*<0.05).

### Effects of Cell Therapy on Foxp3 and STAT5 Expression

**Figure 5:**
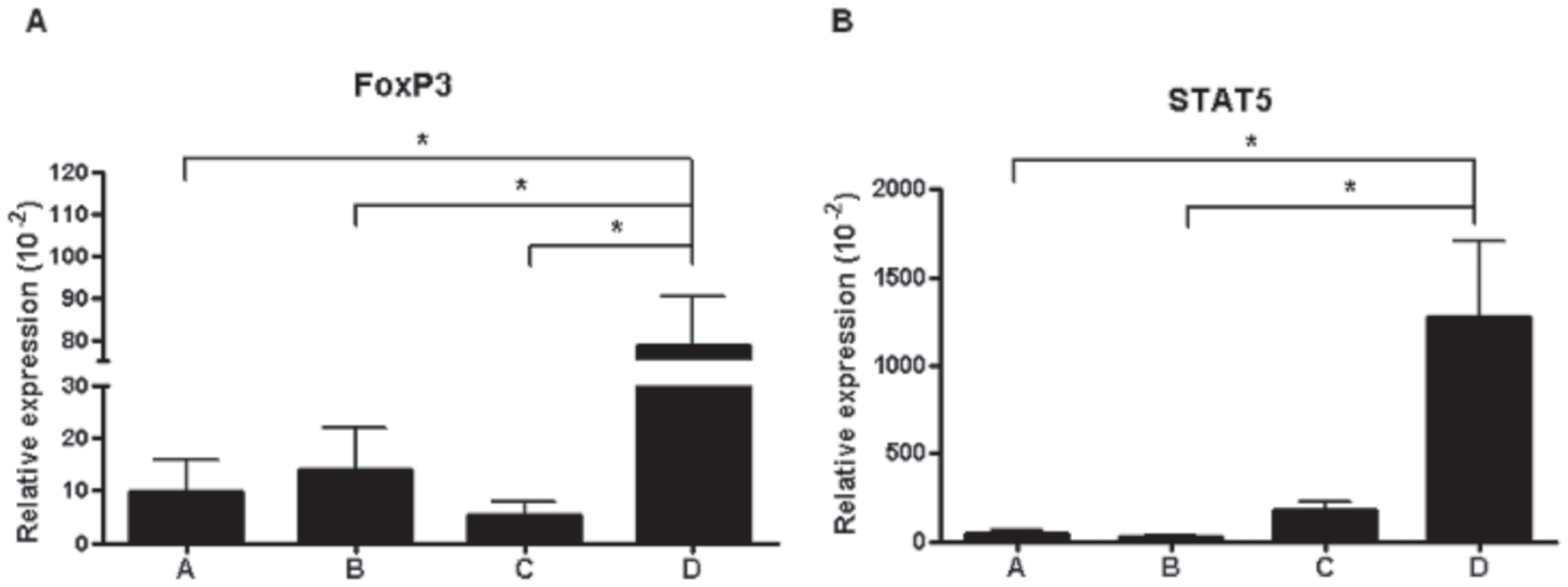
Foxp3 and STAT5 expression profiles. (A) Foxp3 expression in the combination cell therapy group was increased significantly compared to that in the other groups. (B) STAT5 expression in the combination cell therapy group was increased significantly compared to that in other groups. *p<0.05.

Foxp3 expression in the combination cell therapy group was significantly higher than in the control, MSC-treated and Treg-treated groups (*p*<0.05) (Fig. 5), and STAT5 expression in the combination cell therapy group was significantly higher than those in the control and MSC-treated groups (*p*<0.05).

## Discussion

Although many successful CTAs have been performed worldwide, including that of the face, trachea and vascularized bone, the risks associated with lifelong immunosuppression present substantial obstacles to its widespread use [24], [25], [26].

There are three possible options to overcome these issues: (i) a reduction in the toxicity of chronic immunosuppression drugs, (ii) a reduction in the drug dosage by inducing a reduced alloreactive state, and (iii) obviating the requirement for immunosuppression by inducing tolerance, which is the ultimate and ideal alternative [9].

The only way that long-term tolerance has been generated clinically is by induction of mixed chimerism using bone-marrow transplants [27]. However, inducing mixed chimerism (especially, macrochimerism) requires toxic preconditioning that ablates the recipient’s bone marrow. Also, although it is appropriate for haematological malignancies, it would not be an acceptable treatment protocol for skin allotransplantation.

For this reason, generating immunological tolerance by means of an adoptive transfer of tolerance-inducing cells such as MSCs and Tregs, is of growing interest [18], [28], [29], [30], [31]. MSCs produce TGF-β, indoleamine 2,3-dioxygenase (IDO), HGF, PGE2, IL-10 and IL-6, which mediate suppression of the MSC immune response [15], [32], [33]. Additional possible mechanisms of MSC immune suppression include: induction of T lymphocyte anergy and apoptosis; inhibition of mature dendritic cell generation from peripheral monocytes; and induction of Tregs [34], [35], [36], [37].

Despite this, the beneficial effects of MSC-based cellular therapy remain unclear. Recently, Inoue *et al.* [38] found that an MSC infusion failed to prevent allograft rejection. Also, Nauta *et al.* [20] demonstrated that allogenic murine MSCs are not intrinsically immunoprivileged since they can induce a memory T cell response *in vivo*, resulting in graft rejection. Furthermore, Sbano *et al.* [19] showed that allogenic MSCs, when administered to immunocompetent rats, stimulate graft rejection. The immunosuppressive effects of MSCs may be fully understood when the survival of these cells is prolonged by simultaneous administration of immunosuppressive drugs.

For this reason, we speculated that co-infusion of MSCs and Tregs could exert a synergistic immunomodulatory effect. Our results showed that this significantly increased graft survival without the use of postoperative immunosuppressive drugs.

To investigate the underlying mechanisms, the phenotypic features and specific gene (Foxp3, STAT5) expression of splenocytes were evaluated. The results showed that the Th2 subset in the spleen significantly increased in the combination cell therapy group compared with the MSC-treated group while the Th1 subset showed no significant difference between the groups. This shift in the Th1/Th2 balance is important because Th1 cells may be critical in the development of allograft rejection, whereas Th2 cells are involved in promoting graft survival and acceptance [39], [40]. Th2 cytokines are thought to ameliorate the severity of allograft rejection by inhibiting the effects of Th1-mediated cytotoxic T lymphocyte and delayed-type hypersensitivity responses [41]. Also, the total Treg subset increased significantly in the combination cell therapy group compared with the conventional single cell therapy group. Although the origin of this Treg subset (exogenous vs. endogenous) in combination cell therapy group remains to be elucidated in future studies, our results suggest that the increase in the total Treg population mediated tolerance toward allografts. While most previous studies for the role of Tregs in transplantation tolerance have been conducted with recipient derived Tregs, Velasquez-Lopera *et al.* [42] have recently demonstrated that donor-derived Tregs can also significantly enhance skin allograft survival and their actions are donor specific. In addition, there is increasing evidence that the suppressive mechanisms of donor-derived Tregs in transplantation tolerance could be due to the recognition of shared alloantigens of the allograft or a cross-reaction to the mismatched alloantigens of the graft [43]. Similarly, in our study, although we have generated Tregs using non-specific methods, the Tregs could have been alloantigen specific at some point in vivo.

In addition to these differences in T-cell subsets, we found that Foxp3 and STAT5 expression in the combination cell therapy group increased significantly compared to the conventional cell therapy group. Despite many reports that Tregs have an immunomodulatory effect, they may lose their suppressive capabilities *in vivo*, and even differentiate into pathogenic T cells due to their plasticity [44], [45]. Maintaining the *in vivo* stability of Tregs is therefore vital for the development of successful Tregs therapies. Foxp3, a specific Treg marker, is crucial for maintaining the immunosuppressive functions of Tregs [46]. STAT5 expression is also considered to be essential for maintaining Treg homeostasis and self-tolerance [47]. The numbers of Tregs in STAT5-deficient mice were reduced, and transient activation of STAT5 in IL-2-deficient mice increased the numbers of Tregs in the periphery. We believe that this enhanced expression of Foxp3 and STAT5 contributed to the prolongation of graft survival in the combination cell therapy group, by maintaining the stability of the Tregs. In summary, we have shown that a combination cell therapy with MSCs and Tregs has a synergistic immunoregulatory effect in the prevention of skin allograft rejection. However, for a long-term acceptance of grafts, further detailed investigation into the underlying mechanism is necessary, and an optimal protocol is needed, including modification of cell dosage or treatment intervals.
